# Outcomes of Chronic Total Occlusions in Coronary Arteries According to Three Therapeutic Strategies: A Meta-analysis with 6985 Patients from 8 Published Observational Studies

**DOI:** 10.21470/1678-9741-2018-0176

**Published:** 2019

**Authors:** Ying-Ying Zheng, Ying Gao, You Chen, Ting-Ting Wu, Yi-Tong Ma, Jin-Ying Zhang, Xiang Xie

**Affiliations:** 1 Department of Cardiology, First Affiliated Hospital of Zhengzhou University, Zhengzhou, People's Republic of China.; 2 Department of Cadre Ward, First Affiliated Hospital of Xinjiang Medical University, Urumqi, Xinjiang, People's Republic of China.; 3 Heart Center, First Affiliated Hospital of Xinjiang Medical University, Urumqi, Xinjiang, People's Republic of China.

**Keywords:** Percutaneous Coronary Intervention, Myocardial Infarction, Coronary Artery Bypass, *Odds Ratio*, Outcome Assessment (Health Care)

## Abstract

**Objective:**

To perform a systematic review and meta-analysis of studies comparing coronary artery bypass grafting (CABG), percutaneous coronary intervention (PCI), and medical treatment (MT) in patients with chronic total occlusions (CTOs).

**Methods:**

We identified eligible observational studies published in the China National Knowledge Infrastructure database, PubMed, Excerpta Medica database, Google Scholar, Cochrane Library, Web of Science, and "Clinical trials" registration from 1999 to October 2018. Main outcome measures were all-cause mortality, cardiac death, major adverse cardiac events (MACEs), and myocardial infarction (MI).

**Results:**

There were eight observational studies including 6985 patients. Patients' mean age was 64.4 years. Mean follow-up time was 4.3 years. Comparing with MT (2958 patients), PCI (3157 patients) presented decreased all-cause mortality (odd ratio [OR]: 0.46, 95% confidence interval [CI]: 0.36-0.60; *P*<0.001), cardiac death (OR: 0.40, 95% CI: 0.31-0.52; *P*<0.001), MACE (OR: 0.55, 95% CI: 0.43-0.71; *P*<0.001), and MI (OR: 0.40, 95% CI: 0.26-0.62; *P*<0.001). Comparing with MT, CABG (613 patients) presented lower all-cause mortality (OR: 0.50, 95% CI: 0.36-0.69; *P*<0.001) and MACE (OR: 0.50, 95% CI: 0.26-0.96; *P*=0.04), but not lower MI (OR: 0.23, 95% CI: 0.03-1.54; *P*=0.13) and cardiac death (OR: 0.83, 95% CI: 0.51-1.35). Comparing with CABG, PCI did not present decreased risk for those outcomes.

**Conclusions:**

PCI or CABG was associated with better clinical outcome in patients with CTO than MT. PCI is not better than CABG in decreasing mortality, MI, cardiac death, and MACE in coronary CTO patients.

**Table:** 

Abbreviations, acronyms & symbols				
CABG	= Coronary artery bypass grafting		MACE	= Major adverse cardiac events
CAD	= Coronary artery disease		MI	= Myocardial infarction
CI	= Confidence interval		MOOSE	= Meta-analysis of Observational Studies in Epidemiology
CNKI	= China National Knowledge Infrastructure		MT	= Medical treatment
CTO	= Chronic total occlusion		OR	= *Odds ratio*
CTO-SS	= Chronic total occlusion SYNTAX score		PCI	= Percutaneous coronary intervention
EMBASE	= Excerpta Medica database		RCT	= Randomized clinical trial
HF	= Heart failure		UK	= United Kingdom

## INTRODUCTION

Chronic total occlusion (CTO) has been reported to be in approximately 30% of patients with coronary heart disease^[1,2]^. Currently, the management of CTO remains a challenge. Three strategies of management of CTO, including coronary artery bypass grafting (CABG), percutaneous coronary intervention (PCI), and medical treatment (MT), have been usually utilized, but which strategy is the best choice remains controversial. Ladwiniec et al.^[1]^ reported that PCI is associated with improved long-term survival compared with MT alone. Tomasello et al.^[2]^ also reported that PCI significantly improves the survival occurrence in comparison with MT and/or CABG. However, Fujino et al.^[3]^ found out that PCI does not reduce the risk of death or major adverse cardiac events (MACEs), when added to MT. Yang et al.^[4]^ also suggested that PCI did not reduce cardiac death compared with MT in the treatment of CTO. These observational studies and retrospective cohort studies have yielded conflicting results and no large multicenter randomized clinical trial (RCT) has ever tested whether PCI or CABG is superior to MT.

A recent meta-analysis comparing successful *vs*. failed PCI for CTO suggested that successful PCI recanalization of a CTO was associated with improved long-term clinical outcome compared with a failed intervention^[5]^. However, all the participants involved in this meta-analysis have received a CTO-PCI attempt. It is unclear how would be the prognosis of the CTO patients without a CTO-PCI attempt and receiving different management strategy. Therefore, the purpose of this study was to determine if PCI/CABG is associated with improved clinical outcomes compared with the outcomes of MT alone by performing a systematic review and meta-analysis of published studies.

## METHODS

We identified studies published in the China National Knowledge Infrastructure (CNKI) database, PubMed, Excerpta Medica database (EMBASE), Google Scholar, Cochrane Library, Web of Science, and "Clinical trials" registration websites from 1999 to October 2018 using the following keywords: "chronic total occlusions" (CTO); "percutaneous coronary intervention" (PCI); "medical treatment" (MT); "coronary artery bypass graft" (CABG); "coronary artery disease", and "coronary heart disease". The search strategy was as follows: "chronic total occlusions" OR "CTO" AND "percutaneous coronary intervention" OR "PCI" AND "medical treatment" OR "MT" AND "coronary artery bypass graft" OR "CABG" AND "coronary heart disease" OR "CHD" OR "coronary artery disease" OR "CAD".

Main outcome measures were all-cause mortality, cardiac death, MACEs, and myocardial infarction (MI). In the present study, we limited the search criteria to include studies published in the Chinese or English language. Additionally, we also identified studies by searching Clinicaltrials.gov and by hand-searching references cited in relevant publications as described previously^[6]^.

### Data Sources and Study Search Strategy

In the present study, we included observational studies and cohort studies which: 1) enrolled patients with coronary CTO who received treatments of PCI, MT, or CABG; 2) compared the outcomes among treatments of MT, PCI, and CABG; and 3) reported all-cause mortality, MI, cardiac death, and MACE rates.

We excluded: 1) studies assessing the role of different treatment strategies in quality of life; 2) studies comparing outcomes of successful PCI *vs*. failure PCI for CTO unless the outcomes of MT or CABG were also reported; 3) studies that only focused on only one treatment strategy; and 4) studies not involving humans.

### Study Selection

**Fig. 1:**
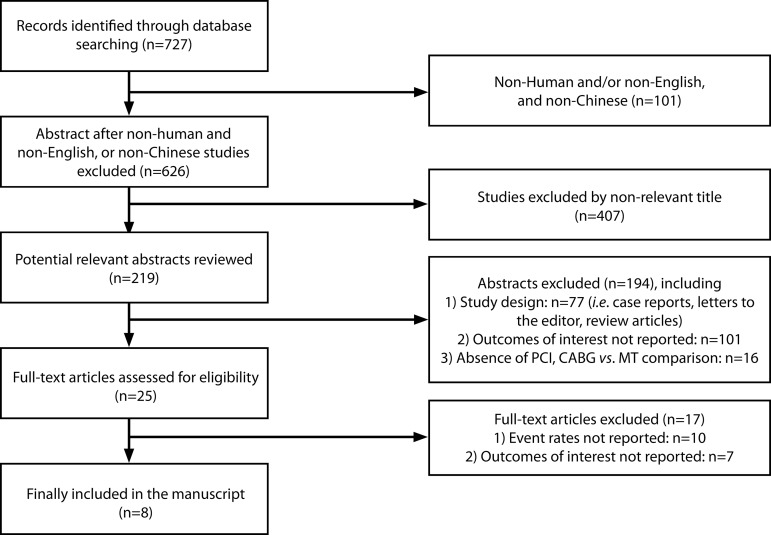
Flow diagram of the literature search and study selection. CABG=coronary artery bypass grafting; MT=medical treatment; PCI=percutaneous coronary intervention

As shown in Figure 1, our initial search yielded 727 citations. Of these, 702 (96.6%) were excluded by title and abstract search because of irrelevant content, non-English and non-Chinese articles, animal subjects, outcomes of interest not reported, or other reasons. The remaining 25 studies were full-text reviewed, and five studies were excluded due to the fact of being case reports or reviews. Furthermore, 12 studies were excluded due to the absence of the interest outcomes reported. Finally, 10 studies^[1-4,7-12]^ met the inclusion criteria, and two studies were further excluded due to duplicated data^[12]^ and no exact data to be used^[7]^.

### Data Extraction

Data were extracted by two investigators (X.X and Y.Y.Z), using standardized data extraction forms. Discrepancies were resolved by consensus. The following contents were collected: name of the first author, year of publication, ethnicity or geographic location of the study subjects, study design, procedural, management strategy, ages, gender, and relevant outcomes.

### Outcomes

The primary outcomes for this systematic review were all-cause mortality and MACE. Secondary outcomes were MI and cardiac death.

### Methodological Quality

We performed this meta-analysis including study selection, data collection, and analysis, and reporting of the results according to the recommendations of the Meta-analysis of Observational Studies in Epidemiology (MOOSE) Group^[13]^.

We calculated weighted *odds ratios* (ORs) and 95% confidence intervals (CIs) for categorical variables. Heterogeneity test was performed using Cochrane Q-statistic and I^2^-statistic^[14]^. Pooled effect sizes were determined using a fixed-effects model (the Mantel-Haenszel method) when heterogeneity was negligible (I^2^ < 50%) or a random-effects model (the DerSimonian and Kacker method) when significant heterogeneity was present (I^2^ ≥ 50%). We also performed a sensitivity analysis to evaluate the effect of each study on the combined ORs by omitting each study in turn. Publication bias was visually estimated by assessing funnel plots and the Begg's test. All analyses were performed using RevMan 5.3 software (Cochrane Collaboration, The Nordic Cochrane Centre, Copenhagen) as described previously^[15,16]^.

## RESULTS

### Studies' and Patients' Characteristics

The characteristics of the eight studies that met eligibility criteria are displayed in Table 1. Of these, one is a prospective cohort study, one is an observational study, and six are retrospective cohort studies. The present analysis includes 6985 patients, of whom 2958 received MT, 3157 received PCI, and 613 received CABG. The mean age of the study participants was 64.4 years. The mean follow-up time was 4.3 years. The overall internal validity was moderate and is illustrated in Table 2.

**Table 1:** Summary of key demographic characteristics of studies included in the meta-analysis.

First author	Publication year	Region	Sample size, n	Management strategy	Follow-up	Age, years	Male sex, %	Outcomes
Fujino et al.^[3]^	2013	Netherlands	820	PCI, MT	7.2 years	-	-	All-cause death, MACEs
Gai et al.^[8]^	2015	China	253	PCI, MT, CABG	5 years	-	-	All-cause death, MACEs, MI, stroke, HF
Ladwiniec et al.^[1]^	2015	UK	1957	PCI, MT, CABG	5 years	CABG:66.0±9.3 PCI: 63.2±10.1 MT: 65.8±10.7	CABG: 82.9 PCI: 73.1 MT: 77.7	All-cause death, MI, cardiac death, repeat revascularization
Tomasello et al.^[2]^	2015	Italy	1777	PCI, MT, CABG	12 months	68.6±11.5	84.1	MACE, MI, cardiac death
Wiggers et al.^[9]^	1997	Denmark	154	CABG, MT	5 years	-	-	All-cause death, MI
Jang et al.^[10]^	2015	Republic of Korea	738	CABG, MT, PCI	3.5 years	MT: 65.6 ±12.0 CABG/PCI: 61.6±10.2	MT: 80.5 CABG/PCI: 83.5	All-cause death, MACE, MI, cardiac death, repeat revascularization
Kim et al.^[11]^	2015	Republic of Korea	393	CABG, MT, PCI	46.5 months	CABG: 61.1±9.6 PCI: 62.0±11.1 MT:67.6±12.6	CABG: 87.0 PCI: 86.9 MT: 79.8	All-cause death, MACE, MI, cardiac death, repeat revascularization
Yang et al.^[4]^	2016	Republic of Korea	1547	MT, PCI	45.8 months	PCI: 65.9±11.3 MT: 61.5±10.8	PCI: 76.7 MT: 80.7	All-cause death, MACE, MI, cardiac death, repeat revascularization

CABG=coronary artery bypass grafting; HF=heart failure; MACE=major adverse cardiac event; MI=myocardial infarction; MT=medical treatment; PCI=percutaneous coronary intervention; UK=United Kingdom

**Table 2:** Analysis of risk of bias.

First author	Publication year	Selection bias	Performance bias	Attrition bias	Detection bias	Multivariate adjustment for possible confounders
Fujino et al.^[3]^	2013	A	B	A	A	Probably adequate
Gai et al.^[8]^	2015	B	B	A	A	Probably adequate
Ladwiniec et al.^[1]^	2015	A	B	A	A	Probably adequate
Tomasello et al.^[2]^	2015	A	B	B	A	Probably adequate
Wiggers et al.^[9]^	1997	B	C	B	B	Probably adequate
Jang et al.^[10]^	2015	A	B	B	B	Probably adequate
Kim et al.^[11]^	2015	A	C	C	C	Probably adequate
Yang et al.^[4]^	2016	A	B	C	D	Probably adequate

This analysis was performed by two independent reviewers. The overall bias of the combined studies was considered low.

A=risk of bias is low; B=risk of bias is moderate; C=risk of bias is high; D=unclear to determine

### All-Cause Mortality

**Fig. 2:**
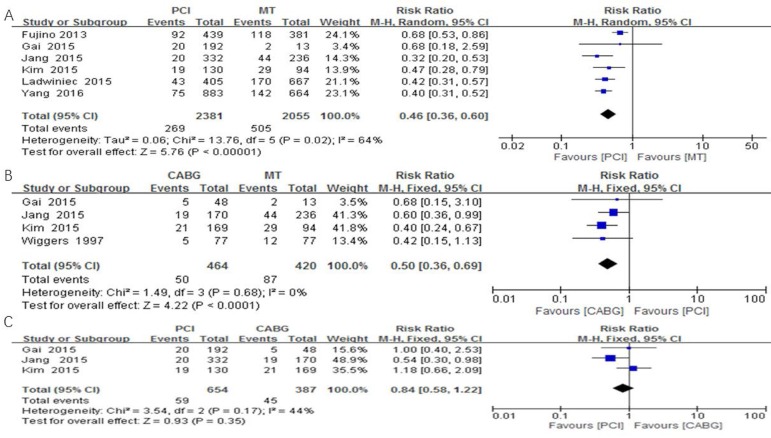
Management strategy and all-cause mortality of chronic total occlusion patients. A=PCI vs. MT; B=CABG vs. MT; C=PCI vs. CABG. CABG=coronary artery bypass grafting; CI=confidence interval; MT=medical treatment; PCI=percutaneous coronary intervention

Of the 6985 patients included in this meta-analysis, 836 (12%) died during follow-up. Comparing with MT, PCI presented decreased all-cause mortality (OR: 0.46, 95% CI: 0.36-0.60; *P*<0.001) (Figure 2A). Similarly, comparing with MT, CABG presented lower all-cause mortality (OR: 0.50, 95% CI: 0.36-0.69; *P*<0.001) (Figure 2B). And we did not find a significant difference between PCI and CABG groups in mortality rates (Figure 2C).

### MACE

Six of eight studies reported 882 MACEs during follow-up. Comparing with MT, PCI presented lower incidence of MACE (OR: 0.55, 95% CI: 0.43-0.71; *P*<0.001). A reduction in MACE incidence was observed not only in the PCI group, but also in the CABG group (OR: 0.50, 95% CI: 0.26-0.96; *P*=0.04). The incidence of MACE in the PCI group did not decrease significantly compared to the CABG group (OR: 0.93, 95% CI: 0.47-1.84, *P*=0.83).

### Myocardial Infarction

Six of eight studies reported 105 MIs during follow-up. Comparing with MT, a reduction in MI was observed among PCI-treated patients (OR: 0.40, 95% CI: 0.26-0.62; *P*<0.001), but not among CABG-treated patients (OR: 0.23, 95% CI: 0.03-1.54; *P*=0.13). However, we did not find a difference in MI between PCI and CABG groups (OR: 1.88, 95% CI: 0.75-4.71, *P*=0.18).

### Cardiac Death

Five of eight studies reported 324 cardiac deaths during follow-up. Comparing with MT, PCI presented lower cardiac death (OR: 0.40; 95% CI: 0.31-0.52; *P*<0.001). We did not find CABG associated with fewer incidence of cardiac death compared to MT (OR: 0.83, 95% CI: 0.51-1.35). Comparing with CABG, PCI did not show advantage in reducing cardiac death risk (OR: 0.45, 95% CI: 0.17-1.22, *P*=0.12).

### Publication Bias Analysis

**Fig. 3:**
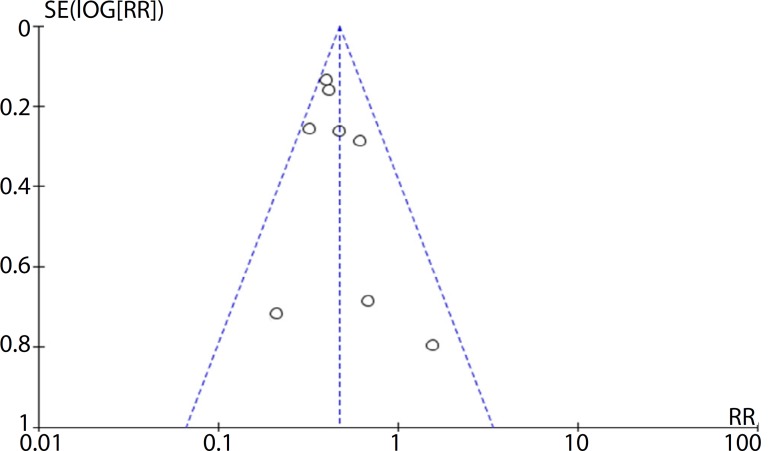
Funnel plot of the association between management strategy and prognosis of coronary chronic total occlusion patients.

In the present study, we utilized funnel plots to evaluate the publication bias of all included studies. No publication bias was identified in this meta-analysis (Figure 3).

### Sensitivity Analysis

**Fig. 4:**
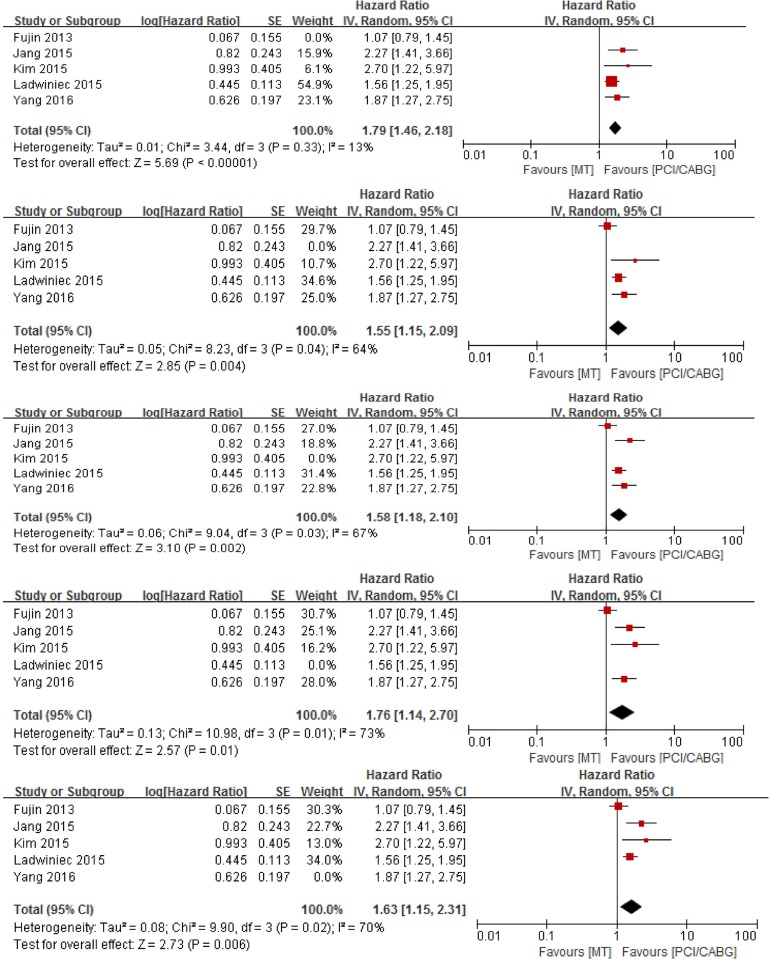
Results of sensitivity analysis. CABG=coronary artery bypass grafting; CI=confidence interval; MT=medical treatment; PCI=percutaneous coronary intervention.

Sensitivity analysis was performed to examine the influence of each study on the pooled ORs by deleting each study one at a time. The pooled ORs showed no significant change (Figure 4), suggesting the results are stable.

## DISCUSSION

The results of this systematic review and meta-analysis of comparison of clinical outcomes among PCI, CABG, and MT in patients with coronary CTO show that PCI presented a 54% reduction in all-cause mortality, a 45% reduction in MACE, a 60% reduction in MI, and a 60% reduction in cardiac death, compared with MT. Similarly, CABG presented a 50% reduction in all-cause mortality and a 50% reduction in MACE, compared with MT. However, compared with CABG, PCI does not have the advantage of decreasing mortality, MI, cardiac death, and MACE in coronary CTO patients. This is the first meta-analysis to compare the clinical outcomes of revascularization *vs*. MT alone in the treatment of coronary CTO patients.

The association between revascularization and low risk for subsequent cardiovascular events may be causal. Revascularization may improve the clinical outcomes of CTO patients by reducing or eliminating myocardial ischemia, which has been linked to worse prognosis^[17]^. At present, our meta-analysis suggested that both PCI and CABG improve the clinical outcomes in comparison with MT. The rates of all-cause mortality, cardiac death, MI, and MACE observed in the MT group were relatively higher than those of the revascularization group (PCI or CABG). Therefore, the findings of the present study have a practical application for cardiologists and surgeons alike. Given the strong clinical benefit in patients with CTO, PCI/CABG may be the optimal management strategies. The incidence of CTOs in the coronary artery disease (CAD) population is from 13% to 24%^[1,18-19]^, however, CTO-PCI was performed in only 5-14% of patients with CTO^[20,21]^. There are several factors which impact the management of CTO patients. Jolicoeur et al.^[22]^ reported that the number of diseased vessels, absence of previous MI, and angina are the strongest predictors of undergoing CTO-PCI. However, following the development of modern techniques and devices for CTO recanalization, the indications are currently increasing. Our meta-analysis' results suggest that PCI/CABG are the best treatment strategies for CTO patients. Furthermore, although our results did not show significant differences in prognosis between CABG and PCI, comparing with MT, PCI presented decreased risks of mortality, cardiac death, MI, and MACE, but CABG only presented decreased risks of mortality and MACE, but not of MI and cardiac death. This fact suggested that PCI rather than CABG might be the best choice for CTO management strategy. However, the revascularization strategy may be influenced by the SYNTAX score and chronic total occlusion SYNTAX score (CTO-SS). In our meta-analysis we did not consider the effect of SYNTAX score and CTO-SS on management strategy selection because they were not provided in the original literatures. Therefore, our results should be further confirmed by future large-scale clinical studies.

### Study Limitations

First, in our meta-analysis, many of the included studies had different entry criteria, study populations, clinical outcomes, and follow-up time. This is a source of increased heterogeneity that may limit the generalizability of our conclusions to the broader coronary CTO population. Second, all the included studies are not randomized trials, therefore, the selection of the treatment group was likely influenced by patients' characteristics and patients' and doctors' preferences. Third, regarding the participants in each study, including both single CTO and multivessel CTO, we did not perform a subgroup analysis according to the number of CTOs, due to the absence of original data. Fourth, the comparison of PCI *vs*. CABG should be interpreted with caution, because the SYNTAX score and CTO-SS were not described in some included studies. Finally, optimization or standardization of MT will affect the clinical outcomes, which should not be underestimated. In our meta-analysis, a stratified analysis of MT was not performed due to the absence of related data in the included studies.

## CONCLUSION

In this first systematic review and meta-analysis of PCI, CABG, and MT in patients with coronary CTO, PCI/CABG were associated with better prognosis than MT. However, PCI is not better than CABG in decreasing mortality, MI, cardiac death, and MACE in coronary CTO patients.

**Table:** 

Authors' roles & responsibilities	
YYZ	Substantial contributions to the conception or design of the work; or the acquisition, analysis, or interpretation of data for the work; final approval of the version to be published
YG	Drafting the work or revising it critically for important intellectual content; final approval of the version to be published
YC	Drafting the work or revising it critically for important intellectual content; final approval of the version to be published
TTW	Drafting the work or revising it critically for important intellectual content; final approval of the version to be published
YTM	Final approval of the version to be published
JYZ	Final approval of the version to be published
XX	Substantial contributions to the conception or design of the work; or the acquisition, analysis, or interpretation of data for the work; final approval of the version to be published
